# The effects of saffron supplementation on inflammatory factors and clinical outcomes in sepsis patients admitted to the intensive care unit (ICU): Study protocol for a double-blind randomized controlled clinical trial

**DOI:** 10.22038/ajp.2024.25221

**Published:** 2025

**Authors:** Shirin Hassanizadeh, Babak Alikiaii, Mohammad Hossein Rouhani, Zeinab Mokhtari, Mohammadreza Moznebiisfahani, Manoj Sharma, Mohammad Bagherniya

**Affiliations:** 1 *Student Research Committee, Isfahan University of Medical Sciences, Isfahan, Iran*; 2 *Nutrition and Food Security Research Center and Department of Community Nutrition, School of Nutrition and Food Science, Isfahan University of Medical Sciences, Isfahan, Iran*; 3 *Anesthesia and Critical Care Research Center, Isfahan University of Medical Sciences, Isfahan, Iran*; 4 *Department of Neurosurgery, Isfahan University of Medical Sciences, Isfahan, Iran*; 5 *Department of Social & Behavioral Health, School of Public Health, & Department of Internal Medicine, University of Nevada, Las Vegas, USA.*

**Keywords:** Critically ill, ICU, Inflammation, Saffron, Oxidative stress, Sepsis

## Abstract

**Objective:**

Research studies have examined saffron's effects on inflammation, infection, and oxidative stress. Nevertheless, the effects of saffron on sepsis patients in the intensive care units (ICUs) have not yet been studied. Hence, this study will examine the effects of saffron supplementation on oxidative stress biomarkers, inflammation factors, and clinical outcomes in critically ill septic patients.

**Materials and Methods:**

Ninety patients with sepsis will participate in this parallel double-blind, randomized clinical controlled trial. In addition to usual care, the intervention group (n=45) will receive a daily tablet containing 100 mg/day saffron for 7 days, and the control group (n=45) will receive a placebo tablet containing 100 mg/day corn starch for the same duration. Acute Physiology and Chronic Health Evaluation II (APACHE II), the Sequential Organ Failure Assessment (SOFA), and the NUTRIC Score will be used to assess the patients' clinical and nutritional status at the beginning and end of the study. Inflammatory markers including erythrocyte sedimentation rate (ESR), C-reactive protein (CRP), interleukin (IL)-6, tumor necrosis factor-alpha (TNF-α), and IL-18, indicators of oxidative stress including malondialdehyde (MDA), glutathione peroxidase (GPx), catalase, superoxide dismutases (SODs), and total antioxidant capacity (TAC), level of Glasgow Coma Scale (GCS), complete blood count (CBC), and lactate dehydrogenase (LDH) will be evaluated at beginning and end of the study. Twenty-eight days after the start of the intervention mortality rates will be assessed.

**Discussion:**

Due to the anti-inflammatory, antioxidant, and antimicrobial effects, saffron might have beneficial effects in critically ill patients with sepsis.

## Introduction

Sepsis is among the most frequent reasons for admission to intensive care units (ICUs) (Genga and Russell 2017). Furthermore, it is one of the leading causes of morbidity and mortality among ICU patients (Sakr et al. 2018). According to recent research, sepsis is a final common cause of infection-related death (Schlapbach et al. 2020). Globally, the incidence of sepsis has been on the rise (Navaneelan et al. 2016). Around 30 million people worldwide suffer from sepsis each year, and more than 6 million people die from it (Organization 2018). In 2011, the United States spent more than $20 million treating septic shock and severe sepsis (Lagu et al. 2012).

 Sepsis is characterized by an uncontrolled and severe immune response to pathogens (bacteria, fungi, parasites, and viruses) (Nedeva 2021). The severity of immune dysfunction and hemostasis problems (coagulation factors and platelets) can lead to cellular and tissue damage, multi-organ failure, and eventually death (Levi and van der Poll 2017). Inflammatory biomarkers and pro-inflammatory cytokines such as interleukin (IL)-6, C-reactive protein (CRP), IL-1, IL-18, and tumor necrosis factor alpha (TNF-α), increase after the development of sepsis (Beltrán-García et al. 2022; Grondman et al. 2020; Mierzchala-Pasierb et al. 2019). Therefore, it is necessary to control inflammation and prevent sudden exacerbation of inflammation and oxidative stress among patients hospitalized in the ICU. 

Despite the availability of antibiotics and fluid resuscitation therapies as effective treatments for sepsis (Brown and Semler 2019), sepsis and its associated complications remain a significant problem in the ICU. Previously, antibiotics have been demonstrated to be insufficient to control the complex mechanism of sepsis (Usmani et al. 2021). In addition, the overuse of antibiotics may result in the emergence of antibiotic-resistant pathogens (Rhee et al. 2020). Based on previous studies, various medicinal plants have the potential to suppress pro-inflammatory and anti-inflammatory cytokines, and the secondary effects of these compounds are minimal or nonexistent (Garnier and Shahidi 2021). Hence, due to the less/no side effects and ease of preparation of medicinal plants, natural compounds have attracted significant interest as an adjunctive therapy to combat the symptoms of sepsis. One of these medicinal plants is saffron (Cardone et al. 2020).

Saffron (Crocus sativus L), a member of the Iridaceae family, is commonly used in food as a flavoring (Khorasanchi et al. 2018). In saffron, more than 150 volatile compounds are present, including carotenoid pigments (crocin, carotene, zeaxan-thin, crocetin, and lycopene), as well as monoterpene aldehydes (safranal and picrocrocin) and monoterpenoids (crocusatines), isophorones, and flavonoids that may contribute to its wide range of biological effects (Asbaghi et al. 2021; Melnyk et al. 2010). Among its active components, particularly crocetin and crocin, are powerful antioxidants and radical scavengers (Puglia et al. 2019). Several studies have revealed the antioxidant, anti-inflammatory, anti-thrombotic, anti-apoptotic, immune system modulatory, and antimicrobial properties of saffron (Asbaghi et al. 2021). Saffron was found to improve erythrocyte sedimentation rate (ESR), CRP, TNF-alpha, malondialdehyde (MDA), and total antioxidant capacity (TAC) levels in rheumatoid arthritis (RA) patients when administered 100 mg daily (Hamidi et al. 2020). The results of a meta-analysis indicated that supplementing with saffron is more beneficial to people with high levels of CRP (Asbaghi et al. 2021).

To our knowledge, no clinical trials have examined saffron's effect on patients with sepsis. This study aims to determine whether saffron supplementation affects inflammatory factors, oxidative stress, and clinical outcomes in patients with sepsis in the ICU. It is hoped that the results of this research can reduce some of the major problems of sepsis patients in the ICU.

## Materials and Methods

**Figure 1:**
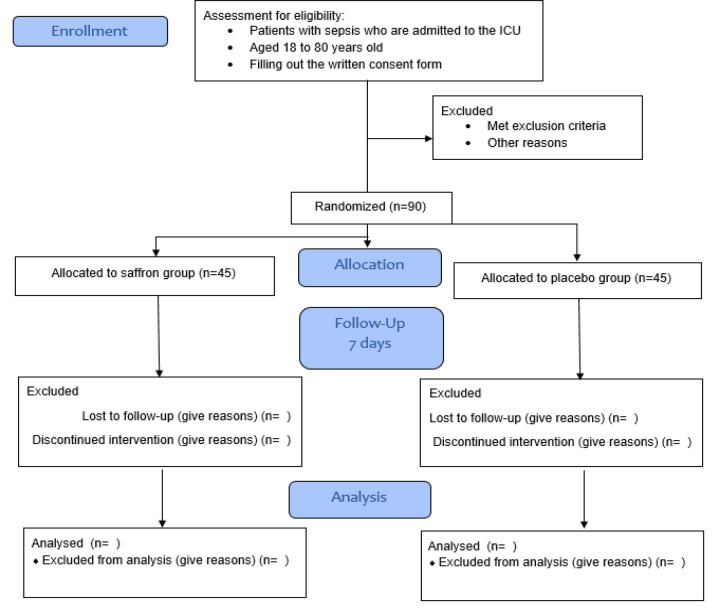
Study Flow Diagram

This study protocol was developed following the Standard Protocol Items: 

Recommendations for Interventional Trials (SPIRIT) 2013 checklist (Chan et al. 2013). It is a randomized, parallel, two-arm, double-blinded, and placebo-controlled clinical trial that will include 90 critically ill patients with sepsis. Figure 1 illustrates the trial design. From May 2024, patients will be selected from the ICU of Al-Zahra Hospital, an academic hospital affiliated with Isfahan University of Medical Sciences, Isfahan, Iran. We will screen subjects according to our inclusion and exclusion criteria before including them in the study. 

### Eligibility criteria

The following criteria will be used to select participants:

### Inclusion criteria

Patients with sepsis who are admitted to the ICU. A diagnosis of sepsis and septic shock will be made using Sepsis-3 definitions by Surviving Sepsis Campaign International Guidelines for Management of Sepsis and Septic Shock (Rhodes et al. 2017) and a confirmation specialist in anesthesiology or infectious diseases. Within 24 hours of diagnosis, septic patients will be recruited. Aged 18 to 80 years old. A legal guardian must write informed consent. Being able to tolerate enteral nutrition and having a normal digestive function. 

### Exclusion criteria

Dissatisfaction of the patient or his legal guardian. Patients who stay in the ICU for <48hr. Patients receiving parenteral nutrition. Patients receiving enteral or oral nutrition initially but transferring to parenteral nutrition due to contraindications. Patients who are incapable of receiving enteral nutrition or who may not be able to receive enteral nutrition in the future as a result of incomplete resuscitation and hemodynamic instability or gastrointestinal disorders such as nausea, persistent vomiting, ileus, obstruction of the intestinal tract, diarrhea that exceeds 500 ml per day, and high-output fistulas. Cancer patients undergoing chemotherapy. Patients suffering from pancreatitis, kidney failure, or congenital or immune disorders. Patients taking phenobarbital, levetiracetam, or phenytoin. Dialysis patients, patients with severe septic shock or sepsis, or DIC (diffuse intravascular coagulation). Pregnancy and breastfeeding. Patients with BMI < 18.5 kg/m^2^. Patients who need blood transfusions frequently. Unwanted side effects associated with taking supplements or placebos. ICU patients who are expected to die within 2 days of admission. Patients who take other herbal supplements. Patients with a spice or herbal supplement allergy. Patients with GCS levels less than 3 or greater than 13. Patients who passed away during the trial

### Randomization

Study participants who meet the inclusion criteria will be enrolled. A random number generator website will be used to randomly assign patients to intervention or control groups: https://www.sealedenvelope.com/simplerandomiser/v1/lists. Patients will be randomized into intervention or control groups with a block size of four (1:1). At randomization, individuals will be stratified by GCS (3 to 8 and 9 to 13) and age (18 to 50 and 51 to 80) so that these variables will be equally distributed between the two groups. The sequence of allocation will be determined by permuted blocks of four. A block size of four creates six different blocks for assigning patients to the trial groups. There will be identical packets and labels for intervention and placebo, each with its own sequence number. One of the investigators who is not involved in patient care will generate the allocation sequence number.

### Intervention

 Both intervention and control groups will receive standard treatments. We will use saffron and placebo (corn starch) as adjunctive therapies. A tablet containing 100 mg of saffron will be given to patients in the intervention group every day. A 100 mg tablet per day of corn starch will be administered to patients in the control group. On each day of the 7-day trial, the placebo or saffron will be administered with enteral nutrition (enteral tube feeding) once a day at 9:00. 

 Saffron and placebo tablets are prepared in packages identical to one another in terms of shape, odor, color, size, and tagged A and B. The saffron powder will be sourced from Mojtahedi Company in Mashhad. Then, experts from the Faculty of Pharmacy at medical University of Isfahan will make the supplement and placebo tablets, and perform a HPLC test. Both groups will receive nutritional support within 24–48 hr of hemodynamic stabilization. The nutritional support will be administered as a bolus method, 7 times in 24 hr, with 25 kcal/kg of energy (Fraipont and Preiser 2013). The patients will receive all commonly prescribed medicines and routine treatment, and undergo daily gastrointestinal monitoring by a physician.

### Blinding

In order to conduct a double-blind trial, A and B will be labeled on the saffron and placebo pill boxes, respectively. Until final analyses are completed, investigators, patients, their legal guardians, laboratory staff, outcome assessors, and data analysts will remain blinded to treatment assignment.

### Ethics approval

Isfahan University of Medical Sciences' ethics committee has approved and accepted the whole protocol (IR.MUI.MED.REC.1402.466). A registration number is available in the Iranian Registry of Clinical Trials (IRCT20201129049534N8). Prior to participating in the study, patients or their legal guardians must fill out the written informed consent form. Throughout the collection process, laboratory specimens, and reports, each patient will be assigned a unique ID number. Research teams will only be able to access the collected data during research and these will remain strictly confidential. Dataset access rights will also be granted to the corresponding author.

### Safety consideration

Several studies have shown that saffron consumption up to 1.5 grams per day is safe in humans (Mehri et al. 2020; Zeinali et al. 2019). Some studies have reported no side effects of saffron, but a small number have reported minor side effects such as dizziness, dry mouth, headache, fatigue, nausea, constipation, and sweating (Zeinali et al. 2019). Hence, at the doses mentioned, saffron and placebo will not cause substantial adverse effects. Nevertheless, minor side effects will be reported to the Isfahan University of Medical Sciences Ethics Committee for consideration.

### Power calculation and sample size estimates

Using the formula for randomized clinical trials, the sample size was calculated considering type I error at 5% and type II error at 20% (β = 0.2; power = 80%). CRP level was considered a main outcome, and based on a previous study, the sample size was calculated to be 35 persons for each group (Δ = 3) (Rai et al. 2017). Considering attrition, 90 patients will be considered in total, 45 in each group.

### Outcome assessment

Our primary outcomes will be serum levels of CRP, TNF-α, IL-6, IL-18, and ESR as a marker of inflammation, serum LDH as an indicator of cell damage, TAC, SOD, GPx, MDA and catalase as an indicator of oxidative stress that will be measured at the beginning and end of the study. In addition, the 28-day mortality rate, GCS, CBC, APACHE II Score, SOFA score, and NUTRIC score are secondary outcomes. Anthropometric variables including weight, height, body mass index (BMI), and calf circumference will be assessed at baseline of the study (Table 1). Mid arm circumference (MAC) will be assessed at baseline and end of the study. 

### Assessment of anthropometric parameters

The patients' height is calculated based on the ulna length due to limitations in the ICU (28):

The patients' weight is calculated based on MAC and Calf Circumference due to limitations in the ICU (29):

### Method of measuring calf circumference

In the supine position, the patients’ leg muscle circumference is measured. The left knee of the patient rises high enough to form a right corner between the thigh and the leg. The tape is placed around the leg muscle and is moved along the muscle to measure the largest circumference without applying pressure to the subcutaneous tissue (Kangalgil et al. 2022).

### Assessment of 28-day mortality

The follow-up of 28-day mortality will be done by phone. 

### Assessment of the level of GCS

Patients' levels of consciousness (GCS) will be extracted from hospital records.

### Evaluation of clinical outcomes

To evaluate the clinical outcomes before and after the intervention, the Acute Physiology and Chronic Health Evaluation II (APACHE II), the Sequential Organ Failure Assessment (SOFA), and the NUTRIC Score will be evaluated (Kalaiselvan et al. 2017; Qiao et al. 2012). To eliminate potential human errors in the calculation of these scores, these scores will be measured by a questionnaire and automatically calculated by a web-based system.

### Laboratory assessment

At the beginning and end of the trial, blood will be drawn. Blood will be collected at 6:00 am before the first gavage to evaluate the serum levels of inflammatory factors (CRP, ESR, IL-6, IL-18, and TNF α), CBC, LDH, and oxidative stress indicators (MDA, catalase, GPx, SOD and TAC). As soon as the blood samples are collected, they will be centrifuged at 3600 rpm, the serum will be separated from the sediment, and it will be preserved at -80°C. Oxidative stress factors will be measured calorimetrically using a commercial diagnostic kit. Measurements of inflammation will be conducted using ELISA kits based on dual biotin antibody sandwich technology. Commercial diagnostic kits will also be used to measure CBC and LDH.

### Statistical methods

SPSS version 22 (SPSS Inc., Chicago, IL, USA) will be used to perform the analysis. For quantitative data, mean and standard deviation (SD) will be reported, and for qualitative data, frequency and percent will be reported. Kolmogorov-Smirnov test will be used to determine whether variables had a normal distribution (Ahad et al. 2011). Paired t-test will be applied to evaluate the differences in each group before and after the intervention. Independent t-test will be used to show the baseline and endpoint differences between the two groups. The Kruskal Wallis test will be used for the between-groups comparison, and the Wilcoxon Ranked Sum test for the inter-group comparisons, if the distribution of data is not normal. We will apply analysis of covariance (ANCOVA) to show differences between two treatment groups after adjusting for confounding variables. For missing data, an intention-to-treat analysis will be performed (Herman et al. 2009). Statistical significance is defined as a p-value less than 0.05.

## Discussion

As far as we know, this is the first clinical trial to investigate saffron's effects on sepsis patients. Despite the increasing prevalence of sepsis in ICU patients and its impact on mortality (Wang et al. 2020), it appears that existing therapies are not effectively controlling the host's response. The current therapeutic approach relies primarily on the timely administration of antimicrobials and supportive treatments such as antibiotics and fluid resuscitation. Nevertheless, overuse of antibiotics can lead to antibiotic-resistant infections (Rhee et al. 2020). In a systematic review, antibiotic overuse was associated with higher in-hospital sepsis mortality (Macias et al. 2022). Hence, the development of new and more effective treatments with minimal side effects is crucial.

The pathophysiology of sepsis is complex, but studies have shown that oxidative stress plays a significant role in sepsis (Arina and Singer 2021). Oxidative stress is caused by an imbalance in the generation of oxidizing free radicals such as superoxide, hydrogen peroxide and hydroxyl ions as well as the removal of antioxidant scavengers like catalase, SOD, and glutathione (GSH), which results in organ damage (Engwa et al. 2022). In several studies, it has been shown that supplementing with saffron can improve these antioxidant markers. An experimental study demonstrated the beneficial effects of crocin and crocetin on improving catalase enzyme levels (Hashemi et al. 2020). An animal study found that saffron administration for 8 days decreased serum LDH levels in rats with MI (Mehdizadeh et al. 2013). LDH is a marker of cellular damage (Sreenivasan et al. 2021), and failure to recover LDH levels within 48 hr can predict mortality (Zein et al. 2004). In another study, 100 mg of saffron per day resulted in significant increases in serum levels of TAC, SOD, and GPx in ulcerative colitis patients (Tahvilian et al. 2021). 

Another factor contributing to sepsis pathogenesis is the inflammatory reactions (Arina and Singer 2021). During an inflammatory reaction, neutrophils and macrophages release cytokines like TNF-alpha, interleukins and prostaglandins. Inhibiting fibrinolysis is one of the functions of cytokines. They activate the extrinsic coagulation cascade (Theofilis et al. 2021). Microvascular thrombosis results from these overlapping processes and organ dysfunction can be caused by thrombosis (Bray et al. 2020). Saffron can reduce inflammatory factors and affect coagulation cascades through various pathways. 

In a randomized, double-blind, placebo-controlled clinical trial, saffron consumption significantly reduced inflammation, especially levels of IL-6 and TNF-a in diabetics (Mobasseri et al. 2020). The consumption of saffron also reduced some blood parameters, including red blood cells, hemoglobin, hematocrit, and platelets (Mehri et al. 2020). Another study assessed 70 ICU patients with sepsis. In this study patients were divided into a control group, which received continuous blood purification treatment, and a treatment group that received continuous blood purification along with SESYA treatment, an extract of saffron known as saffron yellow A. The results revealed that, compared to the control group, the treatment group experienced a significant reduction in serum functional indicators including lactic acid, procalcitonin, CRP, and coagulation function indicators. Additionally, the treatment group demonstrated improved quality of life scores. Both groups showed a substantial decrease in organ function indicators after treatment, with the treatment group exhibiting significantly greater improvement than the control group (Wang et al. 2023). In addition, saffron was shown to improve levels of ESR, CRP, TNF-alpha, MDA, and TAC in patients with rheumatoid arthritis when given at a daily dosage of 100 mg (Hamidi et al. 2020). A meta-analysis study showed that supplementing with saffron can significantly reduce CRP levels in people with CRP levels above 3 (Asbaghi et al. 2021). 

Saffron's medicinal properties can be attributed to volatile and nonvolatile compounds. Safranal, crocin, kaempferol, picrocrocin, crocetin, and a variety of carotenes are among the most important bioactive compounds found in Crocus sativus stigmas (Husaini et al. 2021). Therefore, saffron supplementation appears to be effective in treating sepsis due to its antioxidant, anti-inflammatory, and anticoagulant properties. However, there is still lack of information regarding saffron's potential to improve clinical outcomes in sepsis patients and the underlying mechanisms. To address this critical research gap, this trial will examine how saffron supplementation affects oxidative stress biomarkers, inflammation factors, and clinical outcomes in patients admitted to ICU with sepsis.  

To increase the safety of the intervention, a dose of 100 mg saffron per day will be used, which is completely safe, and previous studies have shown that the use of saffron up to 1.5 grams per day is safe in humans (Mehri et al. 2020).

It should be acknowledged that while this study will be the first randomized double-blind clinical trial to investigate saffron's effects on sepsis, there are some limitations, including the short intervention duration and the lack of long-term follow-up. Additionally, saffron cannot be used as a monotherapy for ethical reasons.

Lastly, if this supplement proves effective in managing sepsis, it could represent a very attractive adjunctive therapy and could have an impact on future medical policy regarding saffron use as an auxiliary treatment for sepsis patients.

**Table 1:** Schedule of enrollment, intervention, and assessment of the clinical trial.

Time line				Study period	
	Enrollment	Allocation	Post-allocation		Follow-up
		Day 1	Day 4	Day 7	28 days after intervention
Enrollment:					
**Eligibility screen**	*				
**Informed consent**	*				
**Randomization**	*				
**Allocation**		*			
Intervention:					
**Placebo (100 mg corn starch)**					
**Intervention (100 mg saffron)**					
**Adverse events**					
Outcome assessment:					
**Biochemical assays**		*		*	
**Assessment of APACHI II score, SOFA score, NUTRIC score**		*		*	
**Assessment of mid arm circumference**		*		*	
**Other anthropometric variables**		*			
**Assessment of mortality**					*

APACHE II: Acute Physiology and Chronic Health Evaluation II, SOFA: Sequential Organ Failure Assessment
